# Public health and corporate social responsibility: exploratory study on pharmaceutical companies in an emerging market

**DOI:** 10.1186/s12992-020-00646-4

**Published:** 2020-12-10

**Authors:** Tatiana Dănescu, Maria-Alexandra Popa

**Affiliations:** 1 „George Emil Palade”, University of Medicine, Pharmacy, Science and Technology of Targu Mures, 38 Gheorghe Marinescu, 540139 Târgu-Mureș, Romania; 2 grid.445667.2 0000 0001 2170 7636 ,,1 Decembrie 1918” University of Alba Iulia, 5 Gabriel Bethlen, 510009 Alba Iulia, Romania

**Keywords:** Pharmaceutical companies’ compliance, Global health, Emerging markets, Corporate social responsibility

## Abstract

**Background:**

Corporate social responsibility (CSR) is studied from many perspectives and has gained unprecedented importance in recent years, especially in emerging economies. Pharmaceutical companies play a very important role in a population’s well-being and health through the CSR and corporate governance practices that they apply.

**Methods:**

We used an exploratory approach to measure compliance with the Corporate Governance Code of pharmaceutical companies listed on the Romanian capital market and with practices declared through CSR.

**Results:**

The results show that pharmaceutical companies are involved in actions that consider the well-being of society by offering financial support and managing various sustainable projects, targeting social and economic issues, leading public health awareness campaigns, and investing in health projects.

**Conclusion:**

This study highlights the increasingly important role played by corporate governance and corporate social responsibility in pharmaceutical companies in improving public health in countries with emerging economies.

## Introduction

Corporate social responsibility (CSR) is defined by the International Organization for Standardization (ISO) as a way in which companies can address and manage social, economic and/or environmental issues for the benefit of communities and as involving actions related to human rights, environmental issues, social inclusion and other related concerns [1]. CSR is a concept that refers to the presumptive debt that companies as social actors have towards all parties involved in achieving their economic activity. Additionally, the role of CSR is to contribute to societal goals, such as philanthropic or charitable objectives, through ethically oriented practices [2], though how adequately this is achieved is debated [3].

In general, our society faces many serious problems such as poverty, disease and environmental destruction, among others. It is proven that some economic systems have undesirable effects on public health [4]. Large corporations have been frequently studied by researchers interested in analyzing their behavior in improving social problems [5]. While such corporations are not legally required to address social issues, the resources available to corporations provide a unique means for solving some of the aforementioned issues [6].. Therefore, CSR implies a high degree of sensitivity from stakeholders but also a willingness to collaborate with parts of civil society that enhance good faith [7, 8].

Pharmaceutical companies harbor a high degree of social responsibility through the products (medicines) that are made available to the general public. Access to these products can make the difference between life and death. Further information and debate regarding how pharmaceutical companies can use their goals and means to undermine the state’s ability to protect the right to drugs are needed [9, 10]. Moreover, a study of several well-known pharmaceutical companies in Europe shows that they exhibit limited transparency in reporting on key aspects of CSR [11]. The results of a study conducted by Demir and Min published in 2019 [12] show that pharmaceutical companies focus on key CSR practices. However, the disclosure of human rights in the analyses of CSR practices by pharmaceutical companies or supply chain information is limited. A study by Fooks et al. [3] analyzes the limits of CSR. They conduct a critical analysis of CSR practices adopted in the tobacco industry and demonstrate that some companies use CSR specific actions that leverage social welfare for profit. This issue is common in developing countries in many industries, where inclusive development is necessary to improve social wellbeing. For example, Frederiksen [13] analyzes, among other issues, the CSR practices of mining companies in Zambia and concludes that they can negatively influence the possibility of inclusive development in exclusionary political settlements.

Other researchers believe that the local community evaluates companies’ CSR practices in terms of their broad effects and not based on the CSR practices transposed into intentional programs designed by corporate management to obtain financial results [14, 15]. However, in the literature, few researchers focus their studies on CSR analysis exclusively [16] while most focus on drug prices and licensing [17, 18] or on evaluating specific CSR activities [19, 20].

CSR has become an important tool for corporate governance quality assurance. The sphere of corporate governance has expanded over time and has become a phenomenon strongly influenced by CSR instruments [21]. Even if the 2008 study by Bondy et al. [22], from the results obtained, cannot claim that codes (of corporate governance, ethics, and conduct) are a primary tool for applying the practices of social responsibility, they observe that, through compliance disclosure statements, corporations also present aspects related to CSR. A study conducted by Flammer et al. [23] published in 2019 shows that adopting CSR as a governance tool is more likely to be effective when CSR practices are numerous. Another study shows that a solid governance structure determines the professional CSR activities defined by the Shin et al. in 2015 as CSR activities undertaken by companies over a long period of time, not individually or tentatively [24]. However, the study does not find a significant influence between governance structures and the voluntary adoption of CSR practices.

CSR has become a business strategy that is well established not only in the academic literature but also in practice. Therefore, it is necessary to examine the motivations behind adopting CSR practices [25, 26]. Under various conditions, firms earn financial benefits [27] or enhance their reputations [28]. A 2005 study analyzes the relationship between corporate philanthropy and shareholder wealth with the author concluding that companies should engage in philanthropic actions to create shareholder benefits [29]. This responsible behavior is stronger for companies with good corporate governance. Another study identifies the importance of a firm’s characteristics for the interests of stakeholders in terms of CSR [30].

Companies submit information on CSR as part of their corporate governance statements together with their financial reports and, in this regard, to declare a positive relationship between their success and their contributions to society. Min et al. [31] study the relationship between CSR and the performance of companies in the pharmaceutical industry. Their results show that the adoption of responsible social behavior positively influences the profitability of pharmaceutical companies.

Romania, as a transition economy, uses economic liberalization as its main engine of economic growth. If economic liberalization has been used by most high-income countries by increasing the competitiveness of the business environment, for emerging economies such as Romania, economic liberalization is more concerned with attracting foreign investment and capital. Some studies demonstrate the importance of foreign capital flows such as foreign direct investments (FDIs) or remittances [32] for economic growth in stimulating savings for recipient households by developing business environments through consumption [33].

The performance of transition economies has been low not only due to large-scale underestimated problems (e.g., accelerated privatization) rooted in perceptions of the economic success of western economies but also due to decision makers’ lack of interest or skills for economic development policies implementation, leading to high inflation and unemployment rates [34]. Therefore, the study of behavior in different sectors of activity in emerging economies represents an area of interest for researchers and specialists. Most studies identified have been conducted in areas such as China or Central and Eastern Europe as the most important areas of the emerging economy in the late 1990s. The risk mitigation capacities of these economies in regard to inflation, corruption, limited transparency, etc. have led to the limitation of foreign investments, and a major cause of these is an absence of effective corporate governance and CSR mechanisms available to protect their investments [35]. Over time, corporate governance codes have been developed to encourage increased transparency in financial reporting to create an attractive capital market internationally.

Emerging economies face many problems related to living standards and inequality, and thus encouraging important developments in other dimensions of sustainable development (through ecological, sustainable and social attitudes) is essential to support economic equilibrium. The World Economic Forum placed Romania among the top 10 of the most inclusive emerging economies in the world in 2018 under its Inclusive Development Index (IDI), which reflects populations’ opinions on their countries’ economic progress [36] in terms of rising living standards and lower wealth inequality. Thus, Romania can serve as a good case for examining the economic and social development practices of emerging markets. The index measures three macroeconomic key-performance indicators: growth and development (measured by GDP and life expectancy), inclusion (including poverty rates and household income) and intergenerational equity (including age demographics and debt per capita).

Romania, as projected by the World Economic Forum, is “advancing” in its IDI through rising living standards. However, its health system is relatively poorly developed. The pharmaceutical industry is central to not only improving quality of life but also supporting the national economy and ensuring the distribution of medicines in the public and private health systems. A study conducted by the Romanian Institute of Economic Forecasting in 2011 [37] shows that the pharmaceutical industry is one of the largest contributors to government revenues, influencing both the national economy and public health. Therefore, we conduct our study on pharmaceutical companies listed on the Bucharest Stock Exchange (BSE), which trade shares regardless of the tier in which it is classified according to BSE [38]. Two of these companies claim, as part of their annual reporting, that they provide and distribute medicines to all public and private hospitals in Romania.

To prove the contributions that pharmaceutical firms make to public health, we consider the requirements imposed on such entities through the Corporate Governance Code (CGC) and the extent to which they are implemented in emerging economies such as Romania. The CGC [39] was developed in 2015 as a result of further requirements of the European Union and replaces the old Corporate Governance Code with both operating according to the ‘apply or explain’ principle. It provides a set of principles and recommendations for increasing the transparency of the financial reporting of companies admitted to the stock exchange. BSE-listed companies, starting in 2016, must disclose information regarding corporate governance through mandatory corporate governance statements including social responsibility actions, pricing policies and other relevant aspects though “apply or explain” principle. Adequate reporting of corporate governance practices increases investor confidence. A statement on corporate governance contains information on compliance with provisions of the CGC and explains deviations from them. Compliance is regularly monitored by the BSE through surveys. In analyzing the pharmaceutical market in Romania, we identify whether the practices adopted by companies are in line with social responsibility requirements imposed by the CGC. The aim is to increase understanding of the importance of corporate governance both among healthcare experts and pharmaceutical companies, thus promoting a broader discussion on the need to encourage CSR practices dedicated to improving population health and social status. We conducted a study for the period of 2014–2018. We contribute to the literature by presenting an exploratory study of an emerging economy that identifies and analyzes CSR practices adopted by pharmaceutical companies with a majority national market share and having a direct impact on population health.

## Materials and methods

Due to the limitations of public financial information for pharmaceutical companies active in Romania, we conducted an exploratory study of all companies listed and operating in the pharmaceutical market - Antibiotice, Medlife, Ropharma, Farmaceutica Remedia, Biofarm, and Zentiva - for which we identified social responsibility practices and calculated CGC compliance levels. These companies are highly influential companies in Romania with diverse ownership structures. Two of the six companies have state capital while the other 4 have majority private capital. The companies’ profiles are presented in Table 1. The entities identified for this study are listed on the BSE’s main segment. Three are listed under the Premium Tier, and the other three are listed under the Standard Tier. According to the BSE, the Premium Tier includes companies with a high liquidity ratio (the top 25 companies) and exhibiting a high degree of transparency and good relationships with investors as measured by the BSE’s own methods. The Standard Tier includes other companies listed on the main segment, but whose shares are not among the top 25 most liquid.

**Table 1:** Profile of BSE listed companies operating on the pharmaceutical market

Company name	Key features in 2019
Antibiotice SA	- holds the leading position in the segment of generic and OTC medicines sold in hospitals and ranks 20th in the overall pharmaceutical market (market share of 1.89%); - is a world leader in the production of the active substance Nystatin; - has majority state capital (over 53%); - exports accounted for 40% of turnover with medicines produced successful in America, Europe and Asia.
Medlife SA	- is one of the largest private medical service companies in Central and Eastern Europe, also being present in the pharmaceutical business, managing 10 pharmacies operating their own clinics; - the market share of the pharmaceutical market is insignificant; - has majority Romanian private capital and is mainly owned by Romanian individuals.
Ropharma	- an important chain of pharmacies that ranks 4th among the top of pharmacy chains operating on the Romanian market with a market share exceeding 7%; - has majority foreign private capital and is mainly owned by foreign legal entities.
Farmaceutica Remedia	- an important chain of pharmacies in Romania with its activities focused on integrated business services such as imports, sales, wholesale distribution, logistics services, marketing, drug registration for major pharmaceutical manufacturers; - its market share of the pharmaceutical market is estimated at less than 3%; - has majority private Romanian capital and is mainly owned by Romanian individuals;
Biofarm SA	- one of the most important producers of medicines and food supplements in Romania, operating mainly on the OTC market; - ranks third on the Romanian pharmaceutical market in terms of turnover; - has majority private Romanian capital and is mainly owned by financial investment companies; - exports represent less than 3% of turnover with products being marketed in 12 countries in Europe and Asia.
Zentiva	- one of the most important drug manufacturers in Romania with production units in Romania and the Czech Republic and ranking second in drug sales on the Romanian market; - has mostly foreign private capital, is part of the Zentiva Group, and is mainly owned by foreign legal entities; - exports represented over 40% of turnover. Until 2018, exports were managed by a company within the Sanofi group.

Source: companies’ audited annual reports for 2019, available at https://bvb.ro

We randomly denote the companies alphabetically with letters A to F as Doppert and Bennett did for their 2015 study.

Documentation was obtained from publicly available audited annual reports that include corporate reports and statements such as: corporate governance statements, CSR documents provided by the entities, annual reports, websites, news and other supplementary materials. Nonfinancial (sustainable) reports were also consulted when identified from the companies’ annual reports or websites. These reports present an integrated view of corporations and include and prioritize sustainability issues relevant to the field and activity of companies, being regulated with the help of European Directive 2014/95/EU [40] and transposed into national law by Order of the Ministry of Public Finance (OMFP) no.1938/2016 [41] and OMFP no. 2844/2016 [42]. According to these regulations, companies with more than 500 employees must produce sustainability reports starting in 2020. Thus, in the period analyzed, some companies voluntarily provided such reports while others disclosed information related to CSR as part of corporate governance sections. In addition, we reviewed the literature to substantiate the study.

From annual financial and/or nonfinancial reports and other documents published by the studied entities, we identified and analyzed social responsibility actions such as social projects that involve employees (blood donations, environmental actions, responsible drug use awareness campaigns, etc.); public health projects; and projects that support the educational, natural, cultural and economic environment.

We determined companies’ CGC compliance levels assuming that the existence of a behavior according to the imposed requirements can guarantee compliance with new CSR requirements that could be developed under the CGC. By 2016, companies were reporting on corporate governance according to the old CGC. Since 2016, the BSE has complied with the international requirements and has adopted the new CGC, which is applicable to all companies listed on the capital market under the Main segment and Premium and Standard tiers. The new Corporate Governance Code replaces the old code and contains more information on the transparency of information, relationships with investors and board of directors related aspects. The analyzed declarations of the conformity of the companies comprise 4 sections with a total of 40 (Standard Tier listed companies) and with 41 (Premium Tier listed companies) subsections presenting requirements that must be met (*N*) and published annually in the corporate governance section of annual reports [42]. To obtain objective, impartial results, we collaborated with an expert in corporate governance, a financial auditor based in Romania, in assigning a weight of importance to each section and subsection (*w*_*i*_) and thus calculate the final compliance level (*CGC*_*weight*_). Based on professional reasoning and experience, the financial auditor assigned to each subsection (requirement) a score (*p*_*i*_) of 0 to 1 as follows: if the pharmaceutical company does not meet the requirement, the score is 0; if the requirement is partially fulfilled, the score is 0.5; and if the requirement is totally fulfilled, the score is 1. The level (weight) of compliance is determined according to the following mathematical formula:

$$ {CGC}_{weight}=\frac{\sum_i^N{p}_i\ x\ {w}_i}{N} $$

The scale of appreciation of the CGC compliance level used by the financial auditor is as follows: a compliance level greater than 75% denotes a high compliance level; a compliance level of between 50 and 75% denotes an average compliance level; and a compliance level of below 50% represents a low compliance level. This scale is widely used in financial and internal audit practice.

The fourth section of the CGC sets out requirements regarding relationships with investors and the social responsibility actions undertaken by a company. The CGC’s main objectives are to increase trust in listed companies and to improve the transparency of reporting and international competitiveness. Therefore, high compliance with the GCC can encourage social responsibility actions, the purpose of which, among others, is also to increase trust. Furthermore, we presented the companies’ turnover share of total turnover in the pharmaceutical market on the BSE. In comparison, we presented the share of CSR reported and identified actions of each analyzed company out of the total number of CSR reported and identified actions in the case of these pharmaceutical companies listed on BSE. Below we present the results obtained.

## Results

To measure compliance with the CGC, we identified “apply or explain” statements for each reporting year, under which entities must report whether they have met requirements recommended by the code and if not, must explain reasons for noncompliance. From scores assigned by the financial auditor, we determined corporate governance statements’ compliance with the GCC both globally and at the level of requirement. The results are presented in Table 2. Corporate governance compliance is calculated based on an assessment of financial auditors’ requirements using the method described above [43].

**Table 2:** Descriptive analysis of corporate governance statements for 2014–2018 period

Analyzed company	Fully/Partially/Non-Implemented Requirements								Calculated conformity rate (%)
	Implemented		Partially		Non-		N/A		
	Nr.	(%)	Nr.	(%)	Nr.	(%)	Nr.	(%)	
A	41	100,00	0	0,00	0	0,00	0	0,00	**100**
B	23	56,10	5	12,20	13	31,71	0	0,00	**47,5**
C	40	97,56	1	2,44	0	0,00	0	0,00	**95**
D	39	95,12	0	0,00	1	2,44	1	2,44	**100**
E	38	92,68	0	0,00	2	4,88	1	2,44	**99**
F	25	60,98	7	17,07	8	19,51	1	2,44	**66,8**
TOTAL	209	–	13	–	24	–	3	–	–
Pharmaceutical industry means	33,60	80,49	2,60	6,34	4,80	11,71	0,60	1,46	**84,72**

Source: authors’ own research and projections based on annual reports published on BSE [43].

Table 2 shows that pharmaceutical companies listed on the BSE fulfill all or some of the 86.83% CGC requirements. We find that companies A and D claim to meet all of the CGC requirements.

A compliance score was calculated for each pharmaceutical company. The highest compliance levels are recorded by companies A and D while company B shows a low degree of compliance. We also calculated the compliance weight of the pharmaceutical industry listed on the Romanian capital market based on individual compliance weights.

We analyze the responsible social behaviors of the pharmaceutical companies for 2014–2018 and briefly present aspects related to actions reported in the entities’ annual reports for local development. Table 3 presents a summary of all social responsibility projects presented in the companies’ annual financial and/or nonfinancial reports during the analyzed period.

**Table 3:** Number of social responsibility projects reported from 2014 to 2018

Social responsibility projects^a^	A	B	C	D	E	F
Public health support (including: blood donations; healthy life promotion; access to medicines; drug safety and side effects;	81	44	70	77	70	56
Social projects and employee management (including: management-employee relationships; anti-corruption measures; supply chains; blood donations; workplace security; open days; etc.)	15	10	11	18	12	8
Educational activities and support (including: financial support for inclusive education; scholarships; open days; training; internships; social and educational projects for local communities, etc.)	5	2	5	5	2	3
Environmental activities and support (including: the efficient use of resources and waste; CO_2_ emissions reduction; green investments; afforestation actions; actions to restore water and public places, etc.)	25	5	10	24	15	5
TOTAL	126	61	96	124	99	72

^a^Note: some projects are assigned to several categories of projects (e.g., blood donation projects are assigned to public health support, social projects and employee management). If a project was developed for several years, it was counted every year, as the results of projects are presented annually in annual reports

Source: authors’ own research and projections based on annual reports published on the BSE

**Fig. 1:**
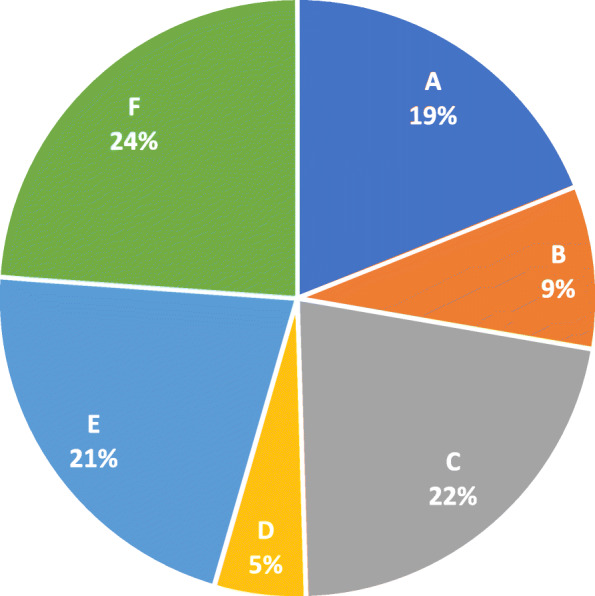
The analyzed companies’ turnover share of total turnover in the pharmaceutical market on the BSE

**Fig. 2:**
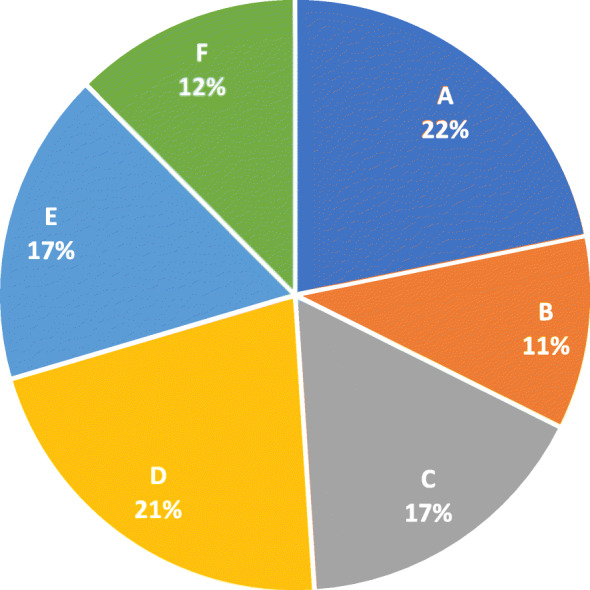
Share of CSR projects reported by each company in the analyzed period

Furthermore, we made a comparison between the share of the companies’ turnover in the total turnover of the pharmaceutical market listed on BVB and the number of CSR projects reported by each company analyzed from the total number of projects reported and identified by the analyzed companies. The results are shown in Figs. 1 and 2.

Companies A, B and C show a similar CSR project share of the turnover share in the total turnover of the pharmaceutical market on the BVB. Company D reports more social responsibility projects relative to the turnover reported for the analysis period, and the other two companies, companies E and F, report fewer CSR projects relative to the turnover share of the pharmaceutical market listed.

## Discussion

To the best of our knowledge, this paper provides the first account of pharmaceutical companies’ commitments to good corporate governance and CSR activities at the national level that can directly influence public health. This study highlights the increasingly important role that corporate governance and corporate social responsibility in the pharmaceutical industry play in improving public health in countries with emerging economies.

We identify some concerns regarding increased transparency in the professional conduct and financial/nonfinancial reporting of pharmaceutical companies listed on the Romanian capital market. Even through the CGC does not require strict compliance with the requirements and the existence of CSR practices has not been regulated until now, pharmaceutical entities tend to comply with and engage in sustainable activities according to the three sustainable development pillars (social, economic and environmental). The studied companies declare that they are involved in solving some community social problems and consider the interests of communities, taking responsibility for employees, shareholders, communities and the environment to ensure the sustainability of these economically and financially strong companies.

The last column of Table 1 shows the CGC compliance level calculated for each pharmaceutical company. The pharmaceutical companies are ordered by compliance level as follows: *A* = *D* ≻ *E* ≻ C ≻ *F* ≻ B. The CGC compliance level mean of the pharmaceutical industry listed on the BSE according to the applied model is 84.72%, reflecting a high level of compliance. Thus, on average, the pharmaceutical industry tends to meet the CGC requirements, indicating a desire to meet the corporate governance requirements.

Adequate and effective governance includes aspects related to CSR, as we find from the CGC requirements. Information on social responsibility is generally included in the fourth section. All six companies analyzed show in this section that they have implemented social responsibility actions. Their assertions are proven by the multiple projects listed or described in the corporate governance section and highlighted in Table 3.

From the annual reports analyzed we observe that in the last years, pharmaceutical companies present a growing interest in sustainable reporting, becoming more involved in specific activities of social responsibility and providing public financial and nonfinancial information on these actions. All six companies have experience in CSR that includes various initiatives (from population health to actions for the economic and social development of the local community and for the involvement of employees in various health programs). However, only one of these companies (company A) discloses a sustainable image through nonfinancial reports and declares 100% CGC compliance. The other pharmaceutical companies disclose information about CSR in their annual financial reports as part of a separate subsection of the corporate governance section. From the results shown in Tables 2 and 3, pharmaceutical companies showing a high CGC compliance level tend to adopt more CSR practices and to disclose more specific information.

The introduction of sustainable reporting and its implementation by pharmaceutical companies on the capital market can enhance the transparency of reporting and credibility and improve a company’s image and, implicitly, its economic performance. Additionally, the emphasis placed on CSR practices by organizations, authorities and the media somewhat force companies to act and comply with positive effects in improving public health and local and regional economic and social development.

While the CGC includes CSR aspects, we believe that the development and addition of new requirements in the CGC referring to CSR aspects together with guidance for implementing the requirements would be useful in encouraging social responsibility practices among companies listed on the capital market regardless of the activity sector. Thus, we present some potential useful aspects in the development of corporate governance practices. In general, CSR actions are intended to develop a company’s image and increase its competitiveness regardless its field of activity. A company with strong socially responsible behaviors can be favored by stakeholders, as they can interpret CSR acts as sincere and transparent. The CSR activities implemented, especially by pharmaceutical companies, must be honest, involving social welfare given the extremely important role that they have in society. Our results show that pharmaceutical companies voluntarily engage in CSR activities involving both employees and local communities.

Given the extent of population health and inclusion problems found in countries with economies in transition such as Romania and how corporations could positively respond to these problems, the following proposals can help improve health and social inclusion: *a)* development of public–private partnerships between pharmaceutical companies and organizations, institutions, authorities and/or other companies that contribute to the improvement of public health (by facilitating access to medicines and the health network, developing health infrastructure, etc.); *b)* authorities granting fiscal facilities and supporting companies that show an interest in responsible behavior; *c)* collaboration with health, social and economic experts to facilitate CSR processes and reporting; and *d)* investing in the sustainable education of the population from the earliest ages to raise awareness of the importance of social responsibility. Although not all our proposals are internationally innovative, in the case of Romania, if adopted, they could improve public health.

## Conclusion

Table 3 shows that pharmaceutical companies are involved in actions that consider societal well-being by offering financial support and consulting for various social projects, targeting entrepreneurial and social environments, conducting awareness campaigns on population health, and investing in health projects. We also identified concerns about the environment, supporting local greening projects, and reducing pollutant emissions and waste produced, which in the long run can reduce risks to population health. We did not find any evidence published regarding violations of ethical norms and social responsibility.

Given the studied companies’ profiles, their implications for economic and social environments, and their unique characteristics, the present study covers an important research area. Our results emphasize the importance of adopting CSR practices to improve population health and social status. We recognize that our results depend on the quality of information presented by the entities’ management in their annual reports. Our results refer to levels of compliance with corporate governance and social responsibility statements and may differ from those of other authors analyzing actual practices identified in the case of pharmaceutical companies. In the case of Romania, we find that pharmaceutical companies tend to declare acts of social responsibility and to corporate governance statements are, to a large extent, complete and compliant with the Governance Code.

The objectives of this exploratory study can be extended to other fields to investigate how the effective corporate governance and CSR practices of companies can influence public health. The expected results could eventually guide strategies and actions for achieving sustainable development goals in emerging economies.

## Limitations

The present article examines the CGC compliance of pharmaceutical companies studied by analyzing the declarations of conformity and sustainability reports (where applicable) without measuring the impacts of actions taken by these companies on public health so our results may differ from other related studies. This paper does not analyze the impact of CSR actions on income or profit obtained from CSR reporting, therefore we consider this a limitation of the research. In our future research, we intend to test the employed practices and to verify their accuracy in accordance with the corporate governance disclosure statements analyzed in this paper.

## Data Availability

Datasets used analyzed are public and can be found consulting the annual reports of the companies, following the external link https://bvb.ro or companies’ websites. Corporate Governance conformity calculation can be accessed at: 10.17605/OSF.IO/NFZ65 .
